# A Systematic Review of the Potential Implication of Infectious Agents in Myasthenia Gravis

**DOI:** 10.3389/fneur.2021.618021

**Published:** 2021-06-14

**Authors:** Victoria Leopardi, Yu-Mei Chang, Andrew Pham, Jie Luo, Oliver A. Garden

**Affiliations:** ^1^Garden and Luo Immune Regulation Laboratory, Department of Clinical Sciences and Advanced Medicine, School of Veterinary Medicine, University of Pennsylvania, Philadelphia, PA, United States; ^2^Research Support Office, Royal Veterinary College, University of London, London, United Kingdom

**Keywords:** myasthenia gravis, autoimmunity, infection, virus, etiology

## Abstract

**Background:** Myasthenia gravis (MG) is an autoimmune disorder of unknown etiology in most patients, in which autoantibodies target components of neuromuscular junctions and impair nerve to muscle transmission.

**Objective:** To provide a synthesis of the evidence examining infectious agents associated with the onset of MG.

**Hypothesis:** We hypothesized that microbes play a pathogenic role in the initiation of MG. For clinical cases, the onset of clinical signs is used as a proxy for the true onset of autoimmunity.

**Methods:** We searched PubMed and Web of Science. Papers captured through database searching (*n* = 827) were assessed, yielding a total of 42 publications meeting the inclusion and exclusion criteria. An additional 6 papers were retrieved from the reference lists of relevant articles. For each pathogen, an integrated metric of evidence (IME) value, from minus 8 to plus 8, was computed based on study design, quality of data, confidence of infectious disease diagnosis, likelihood of a causal link between the pathogen and MG, confidence of MG diagnosis, and the number of infected patients. Negative IME values corresponded to studies providing evidence against a role for microbes as triggers of MG.

**Results:** One hundred and sixty-nine myasthenic patients infected with 21 different pathogens were documented. Epstein-Barr virus (median = 4.71), human papillomavirus (median = 4.35), and poliovirus (median = 4.29) demonstrated the highest IME values. The total median IME was 2.63 (mean = 2.53; range −3.79–5.25), suggesting a general lack of evidence for a causal link.

**Conclusions:** There was a notable absence of mechanistic studies designed to answer this question directly. The question of the pathogenic contribution of microbes to MG remains open.

## Introduction

Myasthenia gravis (MG) is a T cell-dependent, antibody-mediated chronic autoimmune disorder in which autoantibodies attack components of the postsynaptic membrane and impair neuromuscular transmission, resulting in skeletal muscle weakness and fatigue (1). MG is the most common autoimmune disorder of neuromuscular transmission and is estimated to affect ~20 per 100,000 individuals in the United States (2). Pathogenic autoantibodies to muscle nicotinic acetylcholine receptors (AChRs) are found in 85% of patients with generalized MG and 30–50% with ocular MG (3, 4). Half of the remaining patients, who do not have AChR antibodies, have antibodies to muscle-specific kinase (MuSK) (5, 6). The pathogenic role of autoantibodies to AChRs is well-established. These autoantibodies impair neuromuscular transmission by three mechanisms: focal complement-mediated lysis of the postsynaptic membrane that destroys AChRs and disrupts synaptic morphology; cross-linking AChRs by the antibodies, accelerating endocytosis and lysosomal destruction of AChRs; and inhibiting AChR function by direct blockade of AChR binding sites (7–10).

The etiology of MG is largely unknown. Thymic pathologies are frequently associated with MG. The major role of the thymus in the development of MG is supported by the facts that: (1) ~15% of patients with MG have a thymoma; (2) 50% of thymoma patients develop MG; and (3) thymectomy improves the clinical symptoms in some MG patients (11). Another hint of a cause for MG is that some drugs or treatments may lead to MG *de novo*. D-penicillamine, a drug used to treat rheumatoid arthritis, is the prototypical offending drug to induce MG (12). There is growing evidence that immune checkpoint inhibitors, which represent an emerging type of cancer immunotherapy that targets intrinsic down-regulators of immunity, represent a risk factor for progressive MG through an immune-mediated mechanism (13).

Infectious agents have been suspected as a primary external trigger for autoimmune disorders. Multiple studies have reported pathogens associated with MG; however, no study to date has compiled the evidence for infectious agents in the onset of MG in a systematic fashion. Our aim was to compile all the scientific information referring to infectious agents and the onset of MG to interrogate evidence for a causal relationship. All articles that satisfied the inclusion and exclusion criteria were scored using a unique and unbiased equation, yielding integrated metric of evidence (IME) values. Numerical IME values were calculated to assess and categorize the level of evidence supporting the research question.

## Methods

### Literature Review

We searched two databases (Web of Science and Medline) for relevant primary research. Articles captured by the search algorithm [(myasthenia gravis) AND (pathogen OR infectio^*^) AND (virus OR bacteria^*^ parasit^*^ OR fung^*^ OR archaea OR protozoa OR algae OR prion)], denoted as A1, were manually assessed to determine if they met the inclusion criteria, described in Supplementary Table 1. The reference lists of relevant articles were screened to capture any articles not cited in Medline or Web of Science. Additionally, pathogen-specific searches were conducted to further reveal suitable articles.

### Curation of Records

**Figure 1:**
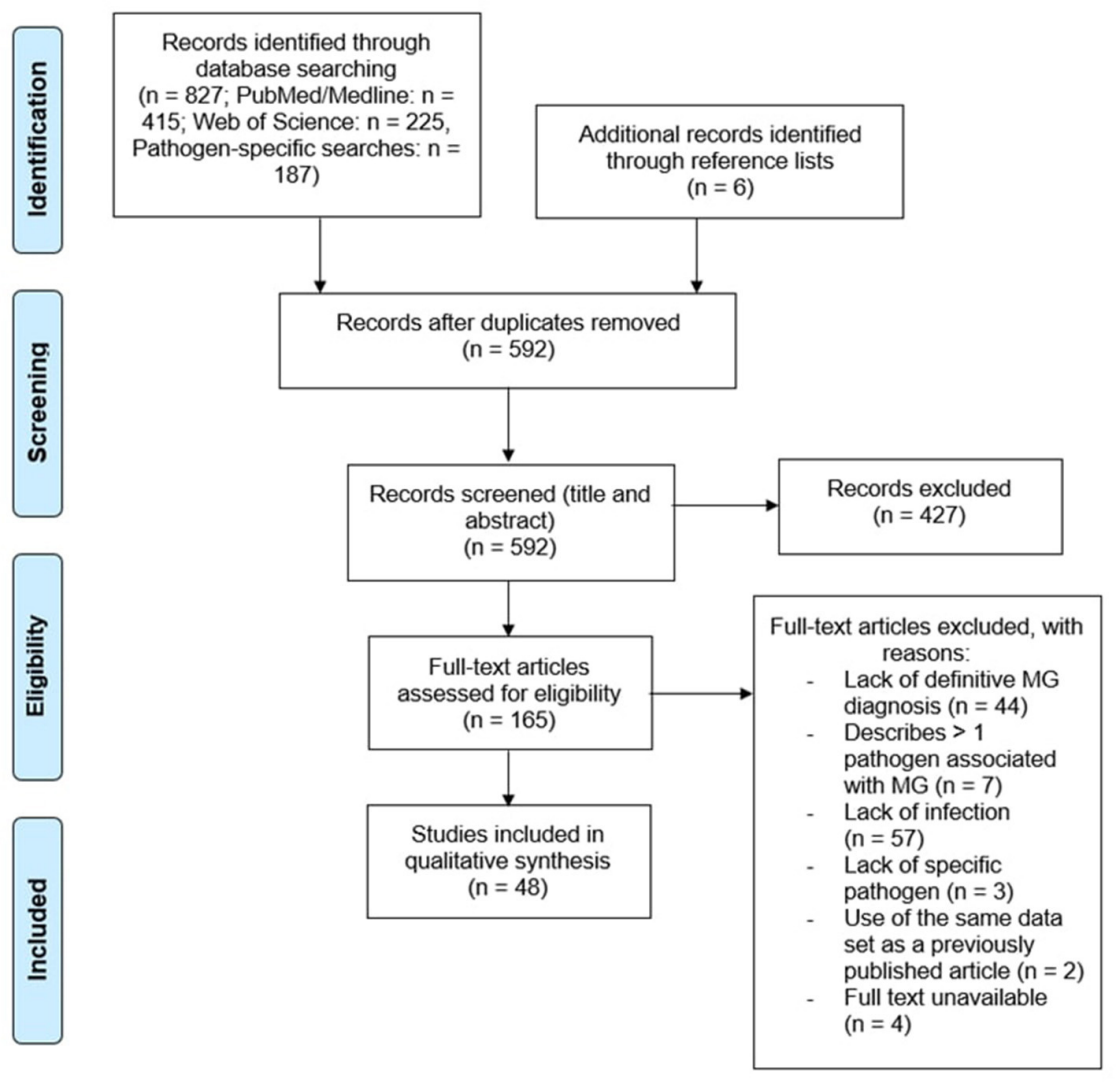
Curation of records. Papers captured by search algorithms in PubMed and Web of Science (*n* = 827) were manually curated to remove duplicates (*n* = 592). Papers were screened to assess whether they met the inclusion and exclusion criteria, yielding a total of 48 publications. An additional 6 papers were retrieved from the reference lists of relevant articles.

A total of 827 papers were captured by search string A1 and pathogen-specific searches. Once duplicates were removed, 592 unique references remained. All abstracts were reviewed, leading to the rejection of 427 records due to their irrelevance to the research topic, data of publication, and/or article type. Papers that did not discuss infectious disease in relation to MG, described infection clearly succeeding MG diagnosis, were published prior to 1978, or were published as review articles were excluded. The cited references in relevant review articles were carefully analyzed to retrieve any publications not captured by the original search. Another 6 papers were retrieved from the reference lists of related articles. Of the 165 papers remaining, an additional 117 records were excluded for the following reasons: a lack of a definitive diagnosis of MG (*n* = 44) as defined in the inclusion criteria, >1 pathogen associated with MG (*n* = 7), lack of infection in myasthenic patients (*n* = 57), failure to describe the specific pathogen associated with MG (*n* = 3), use of same data as previously published work (*n* = 2), or full text unavailable (*n* = 4) (Figure 1).

### Quality Assessment

We developed a unique equation to assess study design (D), the quality of the research paper (Q), the confidence of infectious disease diagnosis (C), the likelihood of a causal link between the infection and MG (L), the confidence of MG diagnosis (I), and, lastly, the number of patients with the given infection (N). For each pathogen identified in a paper, an integrated metric of evidence (IME) was calculated as a sum of normalized scores: IME = 2D + Q + C + 2L + I + N. Each value was weighted according to our assessment of its relevance to evidence rating. The D score determines the accuracy of reported results and conclusions of the paper and L score corresponds directly to the research question. D and L scores were assigned higher weighting in the IME calculation given their relative importance in assessing evidence for a causal relationship between infectious agents and MG. A maximum normalized score of 1 was assigned for each value by dividing the given scores by the highest potential score for the value, yielding a total maximum IME value of 8. A negative score was applied to papers providing evidence against infection as a cause of MG by multiplying the calculated IME value by −1. Individual patients infected with >1 pathogen were not assigned an IME value, and were excluded. The most conservative scores were applied for each criterion to avoid inflation of IME values. Threshold IME values were determined to categorize pathogens as having negligible, low, intermediate, or high evidence for a causal relationship with the onset of MG. The threshold between negligible and low was taken to be a hypothetical retrospective case report, with a Q score of 14, intermediate C, L, and I scores, and 1 positive case (IME = 2.79). The threshold between low and intermediate was taken as a hypothetical cross-sectional study with a Q score of 24, intermediate C, L, and I scores, and 2–5 positive cases (IME = 4.17). Lastly, the threshold between intermediate and high was taken as a hypothetical prospective cohort/case-control study with a Q score of 28, high C score, intermediate L score, high I score (mechanistically based), and 2–5 positive cases (IME = 5.58). Further information on the IME value calculation is provided in Supplementary Table 1.

Ten references were randomly selected from the pool of included articles and independently scored by OAG, VL, and AP. Agreement between reviewers was evaluated using interclass correlation and necessary adjustments were made to the scoring system to avoid misinterpretation. References were re-valued by all reviewers once the necessary changes were made.

### Diagnosis of Myasthenia Gravis

**Figure 2:**
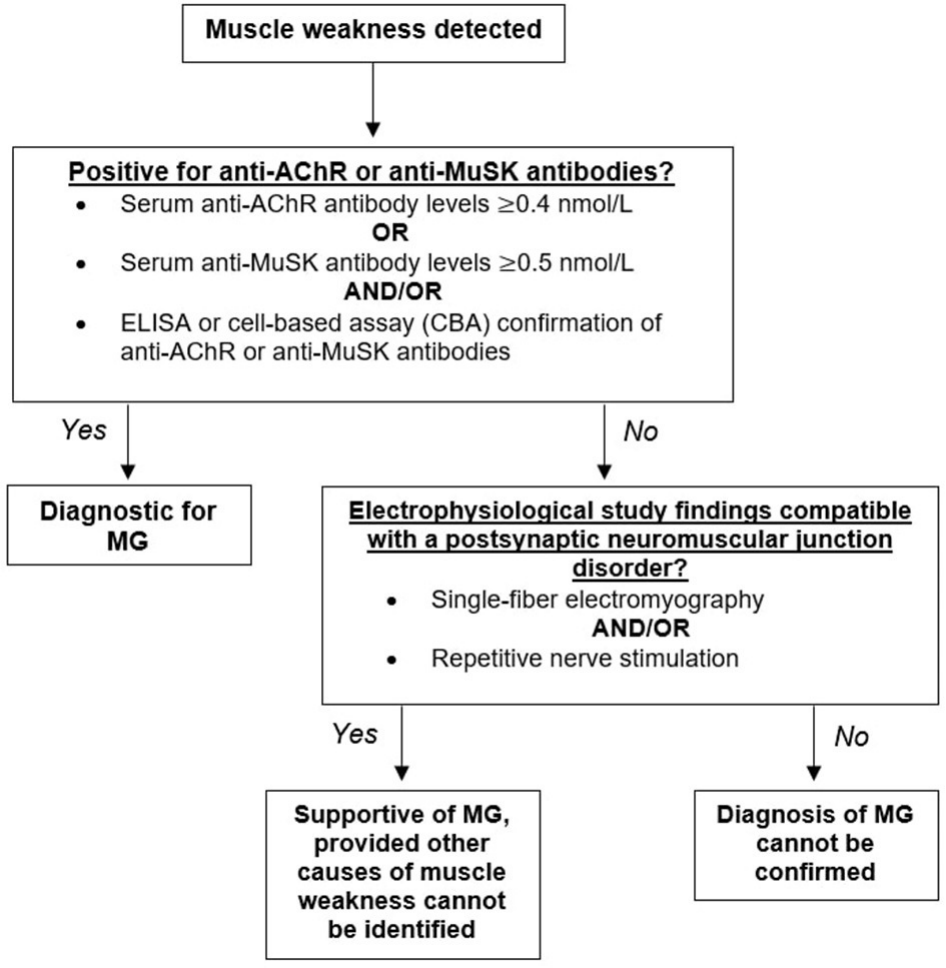
Diagnostic algorithm for myasthenia gravis (MG). Having identified muscle weakness in a patient, biomarkers for myasthenia gravis should next be assessed. Detectible levels of antibodies against the acetylcholine receptor (AChR) or the muscle-specific kinase (MuSK) should be present for a firm diagnosis of MG. Seronegative patients demonstrating muscle weakness confirmed through repetitive nerve stimulation and/or single-fiber electromyography would yield a supportive diagnosis.

The diagnosis of MG is heavily dependent on the history and physical examination of the patient. Muscle weakness or fatigue is often the first indication of disease, which is then followed by a blood test to determine the presence of anti-AChR or anti-MuSK antibodies. Positive detection of anti-AChR or anti-MuSK antibodies through radioimmunoassay, enzyme-linked immunoassay (ELISA), and/or cell-based assays (CBA) is considered diagnostic for MG (Figure 2).

Elevated concentrations of anti-AChR or anti-MuSK antibodies confirms MG diagnosis; however, a normal titer does not exclude disease. A number of patients demonstrating muscle weakness do not have detectable AChR or MuSK antibodies and are classified as “double seronegative.” Repetitive nerve stimulation studies (RNS) and single-fiber electromyography (SFEMG) compatible with a postsynaptic NMJ disorder is supportive of MG, provided that no other causes of muscle weakness can be identified (Figure 2) (14).

## Results

### Agreement of Scoring System

**Figure 3:**
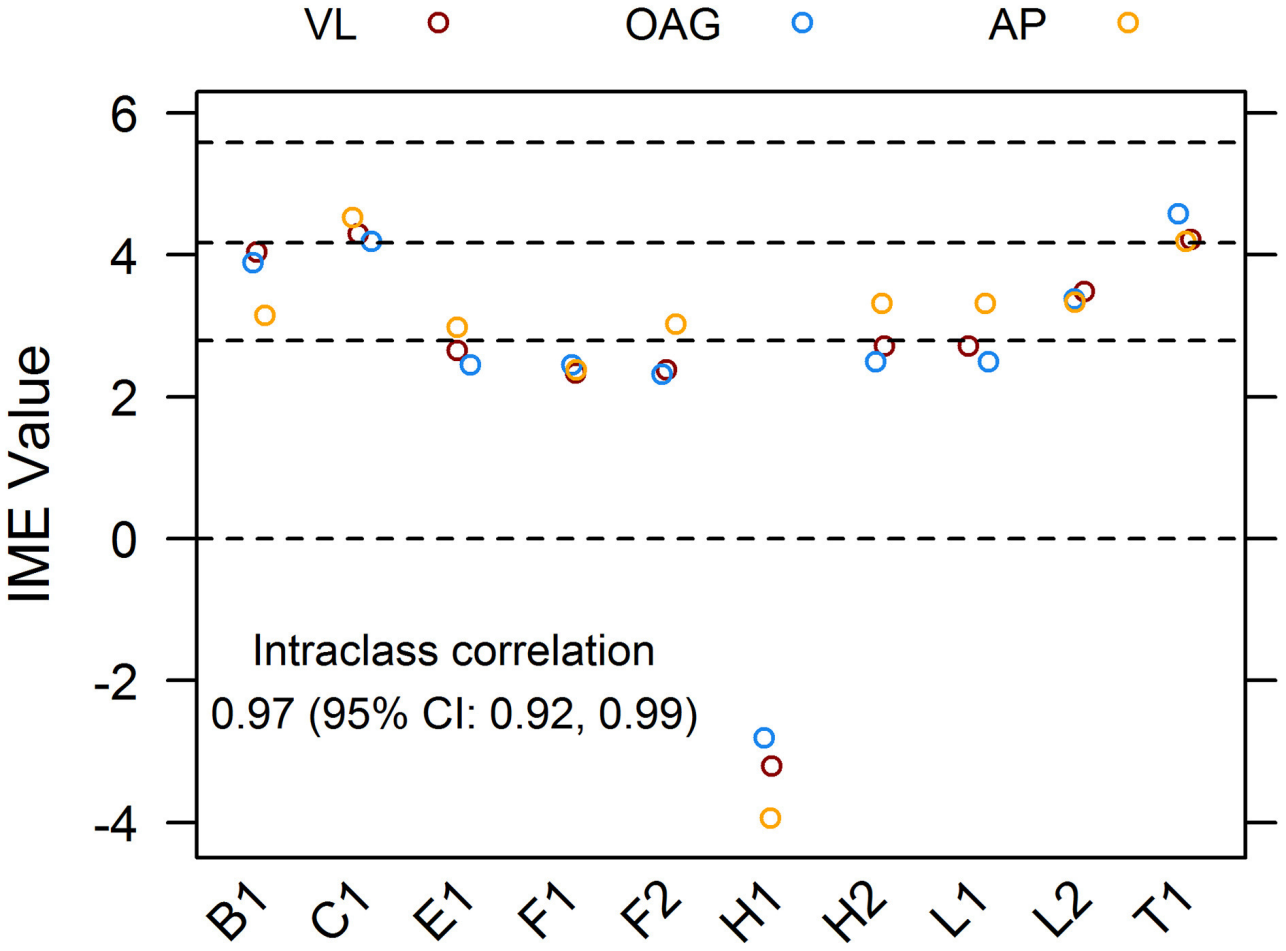
Agreement of integrated metric of evidence (IME) values for ten randomized publications. Ten research papers from the pool of included records were randomly chosen and individually assessed by three reviewers, blind to each other's scores. The interclass correlation between IME values was 0.97, demonstrating excellent reliability of the scoring system. Horizontal dotted lines indicate the threshold IME values between negligible and low (2.79), low and intermediate (4.14), and intermediate and high (5.58) levels of evidence.

To ensure that the qualitative assessment of papers was robust and unbiased, 10 references from the pool of included articles were randomly selected using a random number generator. Papers were scored independently by all reviewers. The intraclass correlation coefficient for the dataset of assigned IME values was 0.97, with 1 being perfect concordance between scores. For the majority, the IME values of individual articles assigned by each reviewer fell within 0.5 points of each other, demonstrating excellent reliability of the scoring system (Figure 3).

### Infection as a Cause of Myasthenia Gravis

**Figure 4:**
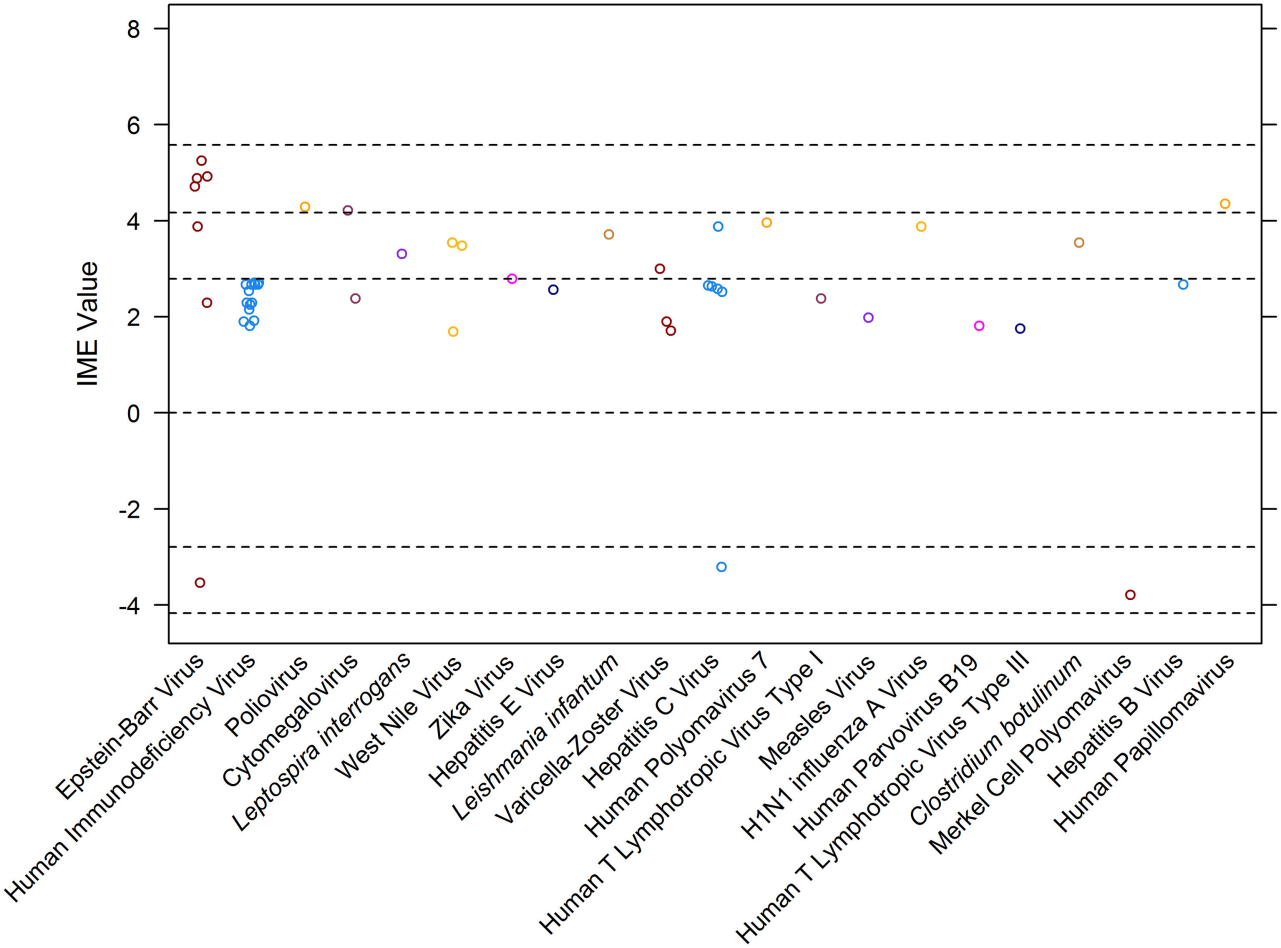
Integrated metric of evidence (IME) values for infectious agents. Horizontal dotted lines indicate the threshold IME values between negligible and low (2.79), low and intermediate (4.14), and intermediate and high (5.58) levels of evidence. The median IME for the dataset is 2.63 (mean = 2.48; range −3.79–5.25).

Overall, 48 manuscripts were reviewed (15–62). IME values were calculated for 21 different infectious agents (Figure 4). Most manuscripts were retrospective case reports/series reporting associations of MG with a specific pathogen. Seven publications, excluded from the calculation of IME, discussed more than one pathogen in association with MG (Figure 1). There was a notable lack of studies directly addressing infection as a cause of MG. Statements are provided for all infectious agents that had at least one study within the intermediate level of evidence range. Human immunodeficiency virus, *Leptospira interrogans*, West Nile virus, Zika virus, hepatitis E virus, *Leishmania infantum*, Varicella-Zoster virus, hepatitis C virus, human polyomavirus 7, human T lymphotropic virus type III, *Clostridium botulinum*, Merkel cell polyomavirus, and hepatitis B virus were all documented in associated with MG, but provided low to negligible evidence supporting a causal link between the pathogen and MG (Figure 4). Derivations of the IME values for all reviewed articles may be found in Supplementary Table 2.

### Epstein-Barr Virus

Seven studies documented 69 cases of MG in patients infected with Epstein-Barr virus (15, 16, 18, 32, 40, 43, 62). The IME values for studies describing Epstein-Barr virus (EBV) ranged from −3.54 to 5.25, with a mean of 3.20 and a median of 4.71. Four out of the 7 studies demonstrated an intermediate level of evidence for a causal link between EBV infection and MG (15, 16, 18, 62). All studies within the intermediate level of evidence range were prospective case-control studies that screened MG patient samples for the presence of infection. The expression of latency protein EBV nuclear antigen 1 (EBNA1) through direct or serological detection was identified in all MG thymic samples (15, 16, 18, 62). One study reported evidence that did not support EBV as a cause for MG; six out of the 16 analyzed early-onset MG thymi demonstrated minimal levels of viral DNA (32).

### Poliovirus

One study documented four MG patients infected with poliovirus (17). The study prospectively analyzed thymic tissue samples from MG patients who underwent therapeutic thymectomies. Out of the 27 MG thymi screened, four revealed the presence of poliovirus RNA and VP1 capsid protein, while all control thymi lacked all indications of infection. Furthermore, poliovirus-infected thymi demonstrated elevated levels of Toll-like receptor 4 (TLR4) activation in cells expressing viral protein VP1, suggesting that the viral infection triggered an inflammatory innate immune response, which could have initiated autoimmunity (17). The IME value for this study was 4.29.

### Cytomegalovirus

Two studies documented MG in patients infected with cytomegalovirus (CMV) (20, 38). The median IME value was 3.29. One reference, a retrospective case report, documented concomitant demyelinating polyneuropathy and MG following CMV infection (38). The patient's MG diagnosis was supported through RNS test, showing decrement in compound muscle action potential; the diagnosis was confirmed by detection of increased concentrations of anti-AChR antibodies. The IME value for this study was 2.38, providing negligible evidence for a causal link between CMV and MG (38). The second study, with an IME value of 4.21, documented 19 cases of CMV infection in MG patients (20). All patients suffered from recently diagnosed late-onset MG and demonstrated T cell receptor Vβ subset expansions of CD8^+^ T cells that correlated with elevated anti-CMV IgG titers (20).

### Human Papillomavirus

One study demonstrated the presence of human papillomavirus (HPV) in 11 patients diagnosed with thymoma-associated MG (54). Thymic tissue samples from thymoma associated patients displayed the presence of viral protein p16, a surrogate marker of HPV infection, and large increases in interferon type 1 (IFN-1) subtypes, IFN-a2, -a8, -& and -b, and Toll-like receptor 3 (TLR3) expression (54). In contrast, control subjects lacked such findings. The IME value for this study was 4.35.

## Discussion

Infections are thought to play a major role in the development of autoimmune disease. Microbes can potentially provoke the immune system to react to self-antigens by molecular mimicry (cross-reacting epitope between the foreign antigen and the host), bystander activation (the activation of autoreactive immune cells by antigenic non-specific mechanisms during an infection), epitope spreading, and viral persistence (polyclonal activation due to the constant presence of foreign antigen driving immune-mediated injury) (63, 64). It has long been suspected that MG can be triggered by infections. However, most articles reporting pathogens associated with MG present negligible to low evidence to support infection as a cause of the disease. A true causal relationship between an infectious agent and MG is further discredited by the likely publication bias toward positive results, emphasizing the lack of evidence supporting infection in the onset of MG. The lack of compelling evidence addressing infection in the etiology of MG is directly related to the lack of mechanistic studies designed to answer the research question.

There is an overall intermediate level of evidence that Epstein-Barr viral infections, particularly within thymic tissue, is associated with the development of MG (15, 16, 18, 32, 40, 43, 62). EBV has previously been linked with the onset of multiple autoimmune disorders, specifically systemic lupus erythematosus, rheumatoid arthritis, and multiple sclerosis, lending support as a potential candidate in the etiology of MG (65). After initial infection, EBV remains in the host in a latent state in a subset of B cells. EBV latency is maintained through the expression of select latent proteins that stimulate the activation, growth, and survival of infected B cells (66, 67). Previous *in vitro* and *in vivo* studies have demonstrated that the production of latent EBV proteins stimulates the expression of *Bcl-2*, an antiapoptotic gene (68). Expression of *Bcl-2* hinders B cell tolerance and is suspected to contribute to autoimmunity by inducing clonal expansion of autoreactive B cell populations (18). The isolation of several latently infected B cells in thymic tissue samples from myasthenic patients supports this hypothesis, but cannot prove causality between EBV and MG (15, 16, 18). The evidence supporting a causal link between EBV and MG is thus speculative at best.

The implications of human papillomavirus and poliovirus in the etiology of MG are each supported by a single study. Both studies measure the presence of the pathogen and markers of inflammation (17, 54). The upregulation of TLR3 and TLR4 occurs in response to pathogen detection and leads to the induction of type 1 IFNs (17, 54). The overexpression of IFNβ, a type 1 IFN, increases the expression of CXCL13 and the AChR α subunit by thymic epithelial cells in both *in vivo* and *in vitro* studies (69). Chemokine CXCL13 is known to mediate recruitment of peripheral B and T cells, and dendritic cells (70). The overexpression of the AChR α subunit in an inflammatory environment in combination with the recruitment of immune cells to the thymus is thought to induce autoimmunity through bystander activation. While both studies demonstrate the upregulation of an inflammatory response to infection, they do not provide a direct link between infection and the onset of MG. Causality between either human papillomavirus or poliovirus and MG has yet to be proven.

Finally, the association between MG and CMV was documented in two studies, with only one falling within the intermediate level of evidence range (20, 38). Myasthenic patients with increased serum concentrations of anti-CMV antibodies demonstrated marked expansions of Vβ CD8^+^ T cells, suggesting a correlation between infection, Vβ CD8^+^ T cells, and MG. While the pathogenic role of CD8^+^ T cells in human MG has yet to be elucidated, *in vivo* rat studies have demonstrated their necessity in this rodent model of MG. CD8-depleted rats were remarkably less susceptible to experimental autoimmune MG than normal rats (71). CD8^+^ T cells are speculated to play a role in the onset of MG through epitope spreading, in similar fashion to that demonstrated in a humanized model for multiple sclerosis (20, 72). Once again, a direct correlation between CMV infection, Vβ CD8^+^ T cells, and the development of MG is lacking.

Overall, multiple pathogens have been associated with the onset of MG, with Epstein-Barr virus, human papillomavirus, and poliovirus presenting the most compelling evidence for a causal link. However, no pathogen has been proven to cause MG and further studies are required to establish the role of infections in the etiology of MG. Observational studies such as prospective cohort and prospective case-control studies, involving larger sample sizes, multiple clinics and institutions, and clear inclusion and exclusion criteria, with appropriate statistical analysis, must be conducted to establish a stronger correlation between a specific pathogen and MG. Mechanistic studies, which provide the highest level of evidence, will ultimately be required to establish causality.

## Data Availability Statement

The original contributions generated for the study are included in the article/Supplementary Material; further enquiries can be directed to the corresponding author/s.

## Author Contributions

VL assessed the relevance of all abstracts and full texts, analyzed and interpreted the results, and wrote the first draft of the manuscript. OG and AP analyzed and assessed a subset of articles to ensure concordance of scores. OG and Y-MC developed the scoring system. Y-MC created the figures. OG and JL conceptualized the idea for the manuscript. OG, JL, and Y-MC edited the manuscript. All authors contributed to the article and approved the submitted version.

## Conflict of Interest

The authors declare that the research was conducted in the absence of any commercial or financial relationships that could be construed as a potential conflict of interest.
