# Supplementary material for: A Systematic Review of the Potential Implication of Infectious Agents in Myasthenia Gravis
Source: Front Neurol. 2021 Jun 14;12:618021. doi: 10.3389/fneur.2021.618021 (PMC8236805; doi:10.3389/fneur.2021.618021)
Supplement: Supplementary file 1 [file Table_1.DOCX]

**Inclusion Criteria**

1. The study reports primary data. Reports of individual cases are suitable if they otherwise fulfill the remaining inclusion criteria.
2. The study is published in complete form in a peer-reviewed journal from 1978 onwards, following the establishment of an autoimmune response against muscle AChR in MG; if written in a language other than English, it will be translated to assess suitability.
3. Myasthenia gravis is diagnosed based on compatible clinical features (i.e. fluctuating fatigability and weakness affecting ocular, bulbar and proximal limb skeletal muscle groups) together with one or more of the following criteria:
   1. Serum anti-AChR antibody levels $\geq$0.4 nmol/L
   2. Serum anti-MuSK antibody levels $\geq$0.5 nmol/L
   3. ELISA or cell-based assay (CBA) confirmation of anti-AChR or anti-MuSK antibodies
   4. Electrophysiological study findings compatible with a postsynaptic neuromuscular junction disorder (repetitive stimulation, single-fiber electromyography, or both)
4. The study describes an infection that precedes the diagnosis of MG or is detected upon MG diagnosis. Studies that describe infections where the onset is unclear in relation to MG will be considered, if they otherwise satisfy the remaining inclusion criteria.

Refined searches conducted in PubMed:

((myasthenia gravis[MeSH Terms]) OR (myasthenia gravis)) AND ((pathogen[MeSH Terms]) OR (pathogen)OR (infectio*) OR (infectio*[MeSH Terms])) AND ((virus) OR (virus[MeSH Terms]) OR (viral) OR (virus[MeSH Terms]) OR ((bacteria) OR (bacteria[MeSH Terms]) OR (bacterial) OR (bacterial[MeSH Terms])) OR ((parasite) OR (parasite[MeSH Terms]) OR (parasitic)) OR (parasitic[MeSH Terms]) OR (fungus) OR (fungus [MeSH Terms]) OR (fungal) OR (fungal[MeSH Terms]) OR (archaea) OR (archaea[MeSH Terms]) OR (protozoa) OR (protozoa[MeSH Terms]) OR (algae) OR (algae[MeSH Terms] OR (prion) OR (prion[MeSH Terms])))

**Integrated Metric of Evidence Calculation and Score Derivations**

For each infectious disease identified in a paper, an integrated metric of evidence (IME) will be computed as follows: IME = 2D + Q + C + 2L + I + N; where study design (D) and quality (Q), confidence of infectious disease diagnosis (C), likelihood of a causal link between pathogen and MG (L), confidence of MG diagnosis (I), and the number of patients with a given infection (N) will be assessed. A maximum normalized score of 1 will be assigned for each value, yielding a total maximum score of 8.

Papers identifying patients infected with >1 pathogen will not be assigned an IME score.

An IME value of 0 will be applied when a study suggests against a causal relationship between the infectious agent and MG

**D score:** A score D, from 1 (lowest) to 6 (highest) will be given based on study design. A “'Retrospective case series or case report” will be assigned 1 point; a “Cross-sectional study” will be given 2 points; a “Retrospective cohort or case-control study” will be assigned 3 points; both “Prospective cohort study” and “Prospective case-control study” will be given 4 points; an “'Unblinded randomized or non-randomized control trial or experiment” will be assigned a score of 5. 'Blinded randomized control trial or experiment” will be assigned the highest score of 6. A normalized D score will be derived by dividing the assigned score by 6.

| **Answer** | **Description** |
| --- | --- |
| Blinded randomized control trial or experiment (prospective) | A blinded randomized control trial (RCT) is one in which cases are allocated at random to receive one of several clinical interventions. One of these interventions is the standard of comparison or control. The control may be a standard practice, a placebo, or no intervention at all. RCTs seek to measure and compare the outcomes after the participants receive the interventions. Since the outcomes are measured, RCTs are quantitative studies. In sum, RCTs are quantitative, comparative, controlled experiments (i.e. prospective in nature) in which investigators study two or more interventions in a series of individuals who receive them in random order. |
| Unblinded, or unrandomized, control trial or experiment (prospective) | As above, but not blinded - i.e. the investigators know which is the experimental and which is the control group. |
| Prospective cohort or case-control study | A cohort study is a quasi-experiment in the form of a longitudinal study (generally a type of observational study). In a cohort study there is a passive follow-up of a group of animals and documentation of relevant characteristics or events related to this group; note that inclusion of information on survival defines a cohort study for our purposes. A cohort study involves an analysis of risk factors and follows a group of animals that do not have the disease, using correlations to determine the absolute risk of developing disease. Cohort studies are largely about the life histories of segments of populations, and the individual animals within these segments. A cohort is a group of animals sharing a common characteristic or experience within a defined period. The comparison group may be the general population from which the cohort is drawn, or it may be another cohort of animals thought to have experienced little or no exposure to the factor under investigation, but otherwise similar. Alternatively, subgroups within the cohort may be compared with each other. Cohort studies may be conducted prospectively, or retrospectively from archived records. |
| Retrospective cohort or case-control study | See above: the study is a cohort or case-control study, but based upon archived records |
| Cross-sectional study | A cross-sectional study is a type of observational study that involves the analysis of data collected from a population, or a representative subset, at ONE specific point in time — that is, cross-sectional data. Cross-sectional studies differ from case-control studies in that they aim to provide data on the entire population under study, whereas case-control studies typically include only individuals with a specific characteristic, comparing them with a sample, often a tiny minority, of the rest of the population. Cross-sectional studies are descriptive studies (neither longitudinal nor experimental). Unlike case-control studies, they can be used to describe not only the odds ratio, but also absolute risks and relative risks from prevalences. They may be used to describe some feature of the population, such as prevalence of an illness, or they may support inferences of cause and effect. At one point in time the subjects are assessed to determine whether they were exposed to the relevant factor and whether they have the outcome of interest. Some of the subjects will not have been exposed nor have the outcome of interest. This clearly distinguishes this type of study from the other observational studies (cohort and case-control), in which reference to either exposure and/or outcome is made. |
| Retrospective case series or case report | A case series tracks subjects with a known exposure, such as animals that have received a similar treatment, or examines their medical records for exposure and outcome. Case series may be consecutive or non-consecutive, depending on whether all cases presenting to the reporting authors over a time period were included, or only a selection. Case series have a descriptive study design: unlike studies that employ an analytical design (e.g. case-control or cohort studies, or RCTs), case series do not involve hypothesis testing to look for evidence of cause and effect, but may be used to speculate on such associations. Case series are especially vulnerable to selection bias: only the presence of a comparator group, which is not a feature of case-series studies, will allow a valid estimate of true treatment effect. Case reports provide information on only a single case. |

**Q Score**: Quality of the articles will be assessed based on the following questions:

- Q1: Is (are) the study hypothesis (hypotheses) that an infectious disease induces (or is associated with) MG clearly stated, OR is the question that an infectious disease induces (or is associated with) MG clearly answered by study design?
- Q2: Is (are) the specific aim(s)/objective(s) of the study clearly stated AND does at least one aim/objective include a means to identify whether an infectious disease induces (or is associated with) MG?
- Q3: Does the study involve multiple hospitals, clinics, and/or research institutions?
- Q4: Does the study include clear inclusion/exclusion criteria?
- Q5: Are there data on the cases screened and excluded from the study?
- Q6: Are the search terms/keywords described?
- Q7: Does the study describe multiple different infectious diseases?
- Q8: Is clinical status clearly defined in relation to infection?
- Q9: Are the results clearly and objectively presented?
- Q10: Are appropriate statistical tests used? (Is data analysis included and are the appropriate statistical tests are implemented for the study design that do not bias the results)
- Q11: Is the measure of variability reported?
- Q12: Is the conclusion supported by the reported results?
- Q13: Is there a clear conflict of interest/disclosure statement?

Answers to Q1-Q6 and Q8-Q13 will be “No/Absent/Unclear/NA” (assigned 0 points), “Partially reported/suggested” (assigned 1 point) or “Yes” (assigned 2 points). For Q7, 2 points will be “All cases are of one type or if diseases of disparate nature were analyzed in separate homogenous groups”, 1 point will be “Stratification is suggested but details are not explicitly stated” and 0 points will be “All other references to disease heterogeneity”.

A weighted sum of these 13 questions will be computed based on the following equation: 2*Q1+2*Q2+Q3+Q4+Q5+Q6+2*Q7+2*Q8+3*Q9+3*Q10+2*Q11+3*Q12+Q13. A normalized Q score will be calculated by dividing the assigned score by 48.

**C Score**: C scores will address the confidence of infectious disease diagnosis. “Direct organism detection (culture, cytology, PCR)” will be assigned a score of 3, “Serological detection of exposure” will be given a score of 2, and “All other signs of infection” will be assigned a score of 1. A normalized C score will be calculated by dividing the assigned score by 3. If multiple methods are used to confirm infection, the least rigorous method used included in the study will represent the allotted score.

| Answer | Description |
| --- | --- |
| Direct organism detection (culture, microscopy, PCR) | Direct detection of organism through culture and/or microscopy, identification of pathogen specific RNA/DNA, or identification of pathogen specific proteins |
| Serological detection of exposure | Identification of antigen specific antibodies |
| All other signs of infection | In the absence of aforementioned diagnostic evidence but infection is still suggested/noted OR the diagnosis is not clear |

**L Score:** L scores will address the causal link between infection and MG. “No/Absent/Unclear/NA” causal link will be assigned a score of 0, a “Suggestive” causal link will be assigned a score of 1, “Supportive” will be assigned a score of 2, and a “Yes/Confirmed” causal link will be assigned a score of 3. A normalized L score will be calculated by dividing the assigned score by 3

| Answer | Description |
| --- | --- |
| Yes | Prospective experimental evidence of causality, which is likely to include mechanistic dissection of immunopathogenesis |
| Supportive | Treatment of infection leads to clinical improvement or remission of MG OR demonstration of immunopathogenic mechanism(s) without prospective experimental interrogation |
| Suggestive | Epidemiological or other inferential (e.g. transcriptomic, immunohistochemical) data |
| No/absent/unclear/NA | All other observations |

**I Score:** I scores will address the confidence of MG diagnosis. “Supportive” will be given 1 point, “Diagnostic” (serological) will be given 2 points and “Mechanistic study” will be given 3 points. A normalized I score will be calculated by dividing the assigned score by 3.

| Answer | Description | Search Term |
| --- | --- | --- |
| Supportive | All other references of MG with a lack of serological evidence OR diagnosis is not clear | “Electrophysiology” OR “Electromyogram” OR “repetitive nerve stimulation” |
| Diagnostic | Positive serological identification of anti-AChR antibodies or anti-MuSK antibodies (titer or ELISA) | “AChR antibod*” OR “Acetylcholine receptor antibod*” OR “Muscle specific tyrosine kinase receptor antibod*” OR “MuSK antibod*” |
| Mechanistic Study | Induction of experimental autoimmune myasthenia gravis (EAMG) | (“Experimental autoimmune myasthenia gravis” OR “EAMG”) AND ((“Mouse” OR “Mice”) OR (“Rat”) |

**N Score:** N score will be given based on the number of patients with the given infectious disease, where 1 point will be assigned for 1 patient, 2 points for 2-5 patients, 3 points for 6-10 patients, 4 points for 11-20 patients, 5 points for 21-50 patients, and 6 points for $\geq$ 51patients. A normalized N score will be calculated by dividing the assigned score by 6.
